# Editorial: Women in brain imaging and stimulation, volume II: 2024

**DOI:** 10.3389/fnhum.2025.1648769

**Published:** 2025-07-08

**Authors:** Molly S. Hermiller, Ruogu Fang, Wen Li

**Affiliations:** ^1^Department of Psychology, Florida State University, Tallahassee, FL, United States; ^2^J. Crayton Pruitt Family Department of Biomedical Engineering, University of Florida, Gainesville, FL, United States; ^3^Louis A. Faillace, MD, Department of Psychiatry and Behavioral Sciences, The University of Texas Health Science Center at Houston, Houston, TX, United States

**Keywords:** brain imaging, brain stimulation, TMS, tDCS, EEG, fMRI

Advancements in brain imaging, neuromodulation, and non-invasive stimulation have significantly enhanced our understanding of brain function, development, and related disorders. While these fields continue to push scientific boundaries, challenges remain in optimizing methodologies, personalizing interventions, and understanding their underlying mechanisms. Addressing such challenges requires diverse perspectives and inclusive research practices to ensure findings translate into effective and equitable clinical outcomes. Since its inauguration in 2022, this Research topic, “Women in brain imaging and stimulation,” has aimed to showcase significant contributions to neuroimaging and stimulation research led by women investigators (Fang et al.). This second volume of “*Women in brain imaging and stimulation*” highlights cutting-edge studies led by women scientists that investigate the complex interactions between brain networks, development, and stimulation, and that demonstrate the critical importance of precision in computational modeling, neuroimaging, and targeted interventions to advance neuroscience research and brain health.

Indahlastari et al. compared current flow models for transcranial direct current stimulation (tDCS) generated using real electrode placements from MRI scans (“real” models) vs. artificially placed electrodes based on virtual 10–20 EEG measurements (“artificial” models) in older adults (mean age = 71.8 years). Significant inverse correlations were found between current density values and brain atrophy in both models, with slightly stronger correlations in the artificial pipeline. Differences in electrode placement between the two models significantly affected current density in key brain regions, although contact area discrepancies did not show significant effects. The findings highlight potential inaccuracies in current density predictions when relying on artificial models. The study underscores the importance of documenting physical electrode placement during tDCS for more accurate modeling and treatment planning.

Wei et al. evaluated the feasibility, safety, and blinding efficacy of a transcranial direct current stimulation (tDCS) paradigm designed to enhance attention by increasing excitability in the dorsal attention network (DAN) and inhibiting the default network (DN; DAN+/DN-tDCS). Forty participants were randomized into either a DAN+/DN-tDCS or sham group and completed a single 20-min stimulation session followed by the Attention Network Test. Results showed significant improvement in executive effect performance in the DAN+/DN-tDCS group but not in the sham group. Notably, blinding was effective, with participants correctly identifying stimulation type at chance levels. The findings suggest DAN+/DN-tDCS enhances attention function and warrant further research into its mechanisms and applications.

Hermiller investigated the effects of continuous (cTBS) and intermittent theta-burst stimulation (iTBS) on network connectivity using resting-state fMRI, focusing on the hippocampal-cortical network. Contrary to the conventional theory that cTBS is inhibitory and iTBS is excitatory, both protocols caused local decreases in connectivity at the stimulated parietal site. cTBS produced both increases and decreases in connectivity within the hippocampal-cortical network, while iTBS primarily caused decreases. cTBS appeared to entrain the hippocampal-cortical network, potentially supporting long-lasting effects observed shortly after stimulation, whereas iTBS failed to maintain this entrainment. These effects were specific to the hippocampal-cortical network, which exhibits an endogenous, functionally relevant, theta rhythm. The findings challenge traditional excitatory/inhibitory models of theta-burst stimulation and highlight the need for further research on its local and network-level impacts.

Mitchell and Nugiel explored how pubertal development, sleep disturbances, and brain network connectivity interact to predict mental health problems in adolescents. Using data from the ABCD Study (N ~ 3,000–10,000), researchers found that advanced pubertal status predicted sleep disturbances. In contrast, both pubertal status and tempo interacted with sleep disturbances to influence mental health outcomes. Three-way interactions between pubertal development, sleep disturbances, and brain network organization also predicted mental health problems. Adolescents with less advanced pubertal status and slower tempo showed stronger links between sleep disturbances, brain connectivity, and mental health issues. The findings highlight the importance of considering these interactions in designing targeted interventions for internalizing and externalizing disorders.

Ho et al. explored the effects of combining transcranial direct current stimulation (tDCS) with exercise on frontal plane kinematics in individuals with patellofemoral pain. The hypothesis was that tDCS targeting gluteal corticomotor function would enhance the impact of hip strengthening exercises compared to sham stimulation. Participants completed two sessions involving either tDCS or sham stimulation during weight-bearing tasks and hip exercises. Frontal plane kinematics and pain (i.e., VAS scores) were assessed pre- and post-session. Results showed no significant improvements in kinematics or pain following a single session of tDCS. The study concludes that while immediate effects were not observed, further research on multi-session tDCS is needed to investigate potential cumulative benefits for patellofemoral pain.

These contributions exemplify the innovative advancements by women scientists in brain imaging and stimulation research, shedding light on critical interactions between brain networks, neurodevelopment, and non-invasive stimulation techniques. They reveal the complex relationships between pubertal development, sleep disturbances, and mental health, while also demonstrating the importance of precise computational modeling and accurate electrode placement for transcranial stimulation efficacy. By investigating cutting-edge non-invasive interventions like tDCS and TMS, these findings challenge traditional paradigms and emphasize the need for targeted, personalized approaches across diverse populations. It is essential that we continue to foster rigorous, inclusive, and collaborative research environments to ensure these discoveries translate into improved outcomes, advancing the fields of neuroscience and brain health for all.

